# Health of the City of London
*Report on the Sanitary Condition of the City of London for 1855 56. By Henry Letheby, M.B. London: 1856.


**Published:** 1857-01

**Authors:** 


### HEALTH OF THE CITY OF LONDON.*

THE total mortality in the City of the year ending Michaelmas is 2,910, or nearly 17 per cent. less than the last year, and about 11 per cent. less than the eight preceding years; "in fact, the death rate of the whole city has been reduced from a general average of 24 per 1000 of the inhabitants, to 22. The death rates differ in proportion in different districts. In the City of London Union, the mortality has been from 15 to 19 in the thousand; in the eastern part, from 22 to 28 in the 1000; and in the southern part of the West London Union, the mortality has risen to 30 in the 1000." The higher mortality of the last named districts depends on the fact that the inhabitants are more firmly fixed to their homes, rather than from the improved sanitary state in the City of London Union. The infant mortality is high. Out of the 2,910 deaths, 38 per cent. were in children under five years of age. This mortality is of course greatest in the crowded alleys and courts of the Eastern and Western Unions. But this is not a fair calculation, for London is not the nursery of its own population. To learn how great is the death rate of infants, the proportion of the dead to the living must be ascertained. In doing this, it will be found that in England the mortality of children under five is about 68 in the 1000, and in country districts only 37. In the city of London it is 82, and in the western division 104.

* Report on the Sanitary Condition of the City of London for 1855 56. By HENRY LETHEBY, M.B. London: 1856.

This represents a mortality 52 per cent. above the average of England, and 181 over that of the country.

The death rate of adults is scarcely less remarkable. Taking the mortality of 15 in the 1000 as natural to this country, it will be found in some parts of the city that this is doubled; and while in England generally the mean duration of life, with men who have reached the twentieth year, is forty years, in the city it is but thirty, and in the western parts only twenty-eight.

Looking at the density of the population of England, we find that there is but one person to every half acre of surface; while in the city there are 179 persons to an acre, and in the western districts 212. This fact, combined with the habits of the people, explains the mortality. The principal fatal diseases are fever, consumption, and infantile disorders.

Dr. Letheby's *Report* is a valuable addition to State Medicine documents.

